# Accuracy and precision of calibrated arterial pulse contour analysis in patients with subarachnoid hemorrhage requiring high-dose vasopressor therapy: a prospective observational clinical trial

**DOI:** 10.1186/cc13715

**Published:** 2014-02-05

**Authors:** Sebastian M Metzelder, Mark Coburn, Christian Stoppe, Michael Fries, Tim-Philipp Simon, Marcus HT Reinges, Anke Höllig, Rolf Rossaint, Gernot Marx, Steffen Rex

**Affiliations:** 1 Department of Anaesthesiology, University Hospital of the RWTH Aachen, Aachen, Germany; 2 Department of Intensive Care, University Hospital of the RWTH Aachen, Aachen, Germany; 3 Department of Neurosurgery Giessen, University Hospital of Giessen and Marburg, Marburg, Germany; 4 Department of Neurosurgery, University Hospital of the RWTH Aachen, Aachen, Germany; 5 Department of Anaesthesiology, University Hospitals of the KU Leuven, KU Leuven, Belgium; 6 Department of Cardiovascular Diseases, KU Leuven, Belgium

## Abstract

**Introduction:**

Calibrated arterial pulse contour analysis has become an established method for the continuous monitoring of cardiac output (PCCO). However, data on its validity in hemodynamically instable patients beyond the setting of cardiac surgery are scarce. We performed the present study to assess the validity and precision of PCCO-measurements using the PiCCO™-device compared to transpulmonary thermodilution derived cardiac output (TPCO) as the reference technique in neurosurgical patients requiring high-dose vasopressor-therapy.

**Methods:**

A total of 20 patients (16 females and 4 males) were included in this prospective observational clinical trial. All of them suffered from subarachnoid hemorrhage (Hunt&Hess grade I-V) due to rupture of a cerebral arterial aneurysm and underwent high-dose vasopressor therapy for the prevention/treatment of delayed cerebral ischemia (DCI). Simultaneous CO measurements by bolus TPCO and PCCO were obtained at baseline as well as 2 h, 6 h, 12 h, 24 h, 48 h and 72 h after inclusion.

**Results:**

PCCO- and TPCO-measurements were obtained at baseline as well as 2 h, 6 h, 12 h, 24 h, 48 h and 72 h after inclusion. Patients received vasoactive support with (mean ± standard deviation, SD) 0.57 ± 0.49 μg · kg^-1^ · min^-1^ norepinephrine resulting in a mean arterial pressure of 103 ± 13 mmHg and a systemic vascular resistance of 943 ± 248 dyn · s · cm^-5^. 136 CO-data pairs were analyzed. TPCO ranged from 5.2 to 14.3 l · min^-1^ (mean ± SD 8.5 ± 2.0 l · min^-1^) and PCCO ranged from 5.0 to 14.4 l · min^-1^ (mean ± SD 8.6 ± 2.0 l · min^-1^). Bias and limits of agreement (1.96 SD of the bias) were −0.03 ± 0.82 l · min^-1^ and 1.62 l · min^-1^, resulting in an overall percentage error of 18.8%. The precision of PCCO-measurements was 17.8%. Insufficient trending ability was indicated by concordance rates of 74% (exclusion zone of 15% (1.29 l · min^-1^)) and 67% (without exclusion zone), as well as by polar plot analysis.

**Conclusions:**

In neurosurgical patients requiring extensive vasoactive support, CO values obtained by calibrated PCCO showed clinically and statistically acceptable agreement with TPCO-measurements, but the results from concordance and polar plot analysis indicate an unreliable trending ability.

## Introduction

Pulmonary arterial thermodilution has long been considered the clinical gold standard for the measurement of cardiac output (CO). Concerns about the inherent risks of pulmonary artery catheterization have driven the development of less invasive devices for monitoring CO [1] of which calibrated arterial pulse-contour analysis (PCCO) as implemented in the PiCCO™-device (Pulsion Medical Systems, Munich, Germany) has become increasingly popular [2]. Meanwhile, the accuracy of pulse-contour derived cardiac output (PCCO) measurements has been tested in a variety of validation studies [3-9]. However, these studies are subject to different limitations. First, the vast majority of validation studies has been performed in cardiac surgical patients receiving if any, only minimal-to-moderate pharmacological hemodynamic support [3-5,9]. Hence, there are a paucity of data on the reliability of PCCO monitoring in situations with significant hemodynamic instability and in settings not related to cardiac surgery. Second, most of the studies assessed the validity of PCCO measurements by analyzing the correlation, bias and the limits of agreement with the chosen reference method [10]. In contrast, the percentage error as an important statistical measure was only reported in a minority of validation studies [11]. Moreover, as the total percentage error is a composite of both the tested and the reference method, a true interpretation of validation studies is only possible if the precision of the PCCO technique and the reference method is described separately [12]. To the best of our knowledge, the precision of PCCO measurements has not yet been reported [7,8].

Furthermore, only a minority of validation studies addressed CO trending, with few investigators reporting concordance rates as an indicator of reliable trending detection [13-15]. No data exist, however, analyzing the concordance between PCCO measurements and a reference method. Recently Critchley *et al*. suggested polar-plot analysis as the most valid method to assess trending ability [16], and this statistical technique has not yet been applied to PCCO measurements.

In the present study therefore, we analyzed the validity of PCCO measurements by comparison with intermittent transpulmonary thermodilution CO (TPCO) measurements. The study was performed in neurosurgical patients who, following the actual guidelines for the treatment of delayed cerebral ischemia (DCI) subsequent to subarachnoid hemorrhage (SAH), were treated with extensive vasoactive support (evidence level: Class I, Level B) [17,18]. In the presence of vasoconstriction, pulse-contour analysis has been recently reported to exhibit insufficient accuracy [19-22]. For the analysis of the agreement with the reference method, we employed the current statistical gold-standard methods, including the separate quantification of the precision of both the PCCO and the TPCO measurements. To investigate the trending ability of PCCO we determined the concordance as recently suggested by Perrino *et al*. [23,24] and additionally employed the polar-plot technique [16].

## Materials and methods

### Patients

After approval by the institutional review board (*Ethik-Kommission an der Medizinischen Fakultät der Rheinisch-Westfälischen Technischen Hochschule Aachen*, EK 171/07) and obtainment of written informed consent by either the patient or legal representative, 20 consecutive patients (16 female and 4 male) were included in this study. The trial was not registered because it was observational and not randomized. All of these patients were simultaneously included in another observational study with a similar study design, comparing the validity of arterial pressure waveform analysis of cardiac output using the FloTrac/Vigileo™-device with TPCO [25]. Patients <18 years of age, pregnant patients, patients from whom written informed consent could not be obtained and patients with occlusive peripheral arterial disease were excluded from the study. All patients suffered from SAH (Hunt and Hess grade I-V) due to rupture of a cerebral arterial aneurysm and subsequent development of cerebral arterial vasospasms. Hypertension induced by high-dose vasopressor support was initiated after the cerebral aneurysm had been interventionally coiled (five patients) or surgically clipped (fifteen patients).

### Patient management

Systemic arterial hypertension was induced by an infusion of norepinephrine to achieve a systolic arterial pressure of approximately 140 to 220 mmHg, resulting in mean arterial pressure (MAP) >100 mmHg and cerebral perfusion pressure (CPP) >80 mmHg (CPP = MAP – intracranial pressure (ICP)) [26]. In addition, all patients underwent a continuous infusion of nimodipine (2 mg·h^-1^). In the event of hemodynamic instability and excessive need of cardiovascular support, the dosage of nimodipine was reduced.

### Hemodynamic monitoring

Routine hemodynamic variables were recorded continuously (Agilent Technologies, Böblingen, Germany). As part of our standard monitoring in these patients, a 5-F thermistor-tipped catheter (PV2015L20A, Pulsiocath, Pulsion Medical Systems, Munich, Germany) was inserted into the femoral artery. In order to monitor and optimize hemodynamic therapy, CO and intrathoracic blood volume (ITBV) were measured by means of intermittent TPCO (PiCCOplus V 5.2.2, Pulsion Medical Systems) [27]. Indicator dilution measurements were performed by quadruple bolus injections of 20 ml of ice-cooled saline 0.9% into the right atrium.

In addition, CO was continuously monitored using arterial pulse wave contour analysis. Pulse-contour analysis was calibrated with transpulmonary thermodilution at predefined time intervals (see below), allowing the calculation of PCCO using the following equation [7]:

(1) $$\mathrm{PCCO}=\mathrm{HR}•\mathrm{cal}•\int_{\text{Systole}}\left(\frac{P\left(t\right)}{\mathrm{SVR}}+C\left(p\right)•\frac{\mathrm{dP}}{\mathrm{dt}}\right)\mathrm{dt}$$

where HR = heart rate; _cal_ = individual calibration factor assessed by transpulmonary thermodilution; $\frac{P\left(t\right)}{\mathrm{SVR}}$ = area under the pressure curve; C(p) = aortic compliance, and $\frac{\mathrm{dP}}{\mathrm{dt}}$ = shape of the pressure curve.

### Hemodynamic measurements

After inclusion of the patients into the study, hemodynamic parameters were assessed at the following time points: inclusion (T_0_), 2 hours (T_2_), 6 hours (T_6_), 12 hours (T_12_), 24 hours (T_24_), 48 hours (T_48_) and 72 hours (T_72_). These time points also served to recalibrate PCCO measurements with transpulmonary thermodilution. Of note, only PCCO values recorded immediately prior to recalibration of pulse-contour analysis were included in the analysis of validity.

### Statistical analysis

Statistical analysis was performed using Sigma Plot (Sigma Plot® for Windows Version 11.0; Systat Software Inc. Chicago, US). All data are expressed as mean ± SD unless indicated otherwise. After testing for normal distribution using the Shapiro-Wilk test, normally distributed hemodynamic variables were compared with baseline by analysis of variance (ANOVA) for repeated measurements [28,29]. Non-normally distributed data (CVP, ITBV and vasopressor doses) were analyzed using Friedman ANOVA. If the analysis of variance revealed a significant interaction, post hoc analysis and correction for multiple comparisons were performed using the Tukey honest significant difference (HSD) test.

Linear regression analysis was used to describe the relationship between TPCO and PCCO measurements, both for absolute values and for percentage changes in CO, as well as for the dependency of PCCO measurements from systemic vascular resistance (SVR).

Bias and limits of agreement were calculated according to Bland and Altman [10] and adjusted for repeated measurements assuming a non-constant situation according to the procedure originally described by Bland and Altman [30]. Bias was defined as the mean difference between TPCO and PCCO values and the limits of agreement were calculated as the bias ± 1.96 SD: 95% of the differences between the two methods were expected to lie within this range.

According to Critchley and Critchley, for comparison of CO values [11] the percentage error (PE) was calculated as follows:

(2) $$\mathrm{PE}=\frac{1.96•\mathrm{SD}}{\mathrm{meanTPCO}}•100\left[\%\right]$$

To assess the accuracy and precision of the reference method we calculated the coefficient of variation as:M

$$\left(\mathrm{CV}=\frac{\mathrm{SD}}{\mathrm{meanTPCO}}\right)$$

and the coefficient of error as:

$$\left(\mathrm{CE}=\frac{\mathrm{CV}}{\sqrt{n}}\right)$$

of the quadruple TPCO measurements for each timepoint as suggested by Cecconi *et al*. [12]. Therefore, the precision of the PCCO measurements was determined using the following equations [12]:

(3) $$CV_{\mathrm{TPCO}-\mathrm{PCCO}}=\sqrt{\left[{\left(CV_{\mathrm{TPCO}}\right)}^{2}+{\left(CV_{\mathrm{PCCO}}\right)}^{2}\right]}$$

where

CV_TPCO-PCCO_ = CV of the distinctions between the two methods;

CV_TPCO_ = CV of TPCO measurements;

CV_PCCO_ = CV of PCCO measurements,as:

Precision_TPCO_ = precision for the reference method = 2 CE_TPCO_

Precision_PCCO_ = precision for PCCO = 2 CV_PCCO_

PE_TPCO-PCCO_ = Percentage error known from the Bland-Altman-Plot (= 2 CV_TPCO-PCCO_)then:

(4) $$PE_{\mathrm{TPCO}-\mathrm{PCCO}}=\sqrt{\left[{\left(\mathrm{Pr}\mathrm{ecisio}n_{\mathrm{TPCO}}\right)}^{2}+{\left(\mathrm{Pr}\mathrm{ecisio}n_{\mathrm{PCCO}}\right)}^{2}\right]}$$

and ultimately:

(5) $$\mathrm{Pr}\mathrm{ecisio}n_{\mathrm{PCCO}}=\sqrt{\left[\left(PE_{\mathrm{TPCO}-\mathrm{PCCO}}\right)-{\left(\mathrm{Pr}\mathrm{ecisio}n_{\mathrm{TPCO}}\right)}^{2}\right]}$$

According to the recommendations originally proposed by Critchley and Critchley [11] the acceptance of the PCCO method is to be judged against the ± 10 to 20% accuracy of the reference method (that is, TPCO). Consequently, limits of agreement between PCCO and TPCO of <30% are to be accepted.

Two consecutive measurements using the same method offer the possibility to quantify the absolute and percentage change within the measured parameter, here, ΔCO. To assess the reliability of trending detection of the test method compared with the reference method, we used the method first described by Perrino *et al*. in 1994, that is, based on regression analysis, direction of change statistics and concordance [24]. Concordance is the agreement of the direction of change obtained from paired measurements of both the test and the reference method. The concordance was assessed by plotting the test ΔCO against the reference ΔCO on a four-quadrant scatter plot. The concordance rate is the percentage of the number of data points lying in the upper right and the lower left quadrant of the scatter plot in relation to the total number of data points. Data at the center of the plot represent only small and random changes in CO and hence were excluded from the analysis (exclusion zone). According to the recommendations recently proposed by Critchley *et al*., in the present study we used an exclusion zone of 15%, and sufficient concordance to assume interchangeability was set to >90 to 95% [16].

Last, we applied the polar plot technique to analyzed trending ability as recently described in detail by Critchley *et al*. [16]. Briefly, the four-quadrant plot as used in the concordance analysis presents the ΔCO data as a cartesian (x, y) vector that has both direction and magnitude. By converting the x-y values to polar coordinates, a new polar plot can be drawn that shows agreement as the angle θ (angle made by ΔCO vector with the line of identity (y = x)) against the mean change in CO as the radian (distance of the data point from the center of the polar plot). The better the agreement between CO measurements, the closer the data pairs will lie along the horizontal radial axis (that is, within 10% of mean CO, limits of agreement).

## Results

**Figure 1:**
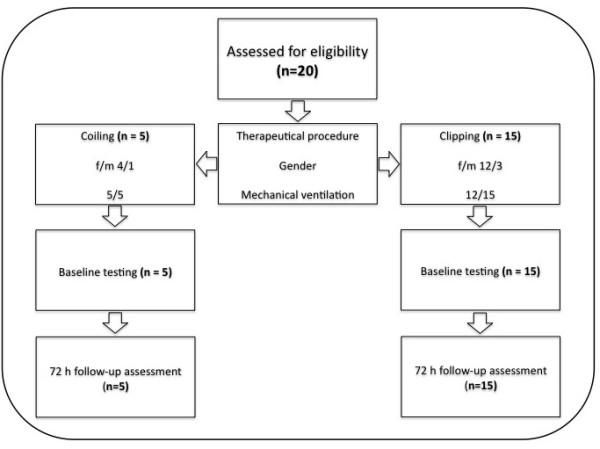
Flow chart of the included patients.

A total of 20 patients were included in this study (Figure 1). Demographic and biometric data of the included patients are presented in Table 1. Hemodynamic data obtained at each time point are depicted in Table 2. Except for MAP at T_48_, there were no significant changes during the observation period.

**Table 1:** Demographic and biometric data

	
Gender, female/male	16/4
Age, years	45 ± 8
Height, cm	171 ± 7
Weight, kg	74 ± 12
BSA, m²	1.86 ± 0.16
Time of onset of vasospasm, days (median/range)	5 (3 to 13)
Hunt and Hess grade (median/range)	4 (2 to 5)
Therapeutic procedure, clipping/coiling	15/5

Data are presented as mean ± SD if not otherwise indicated. BSA, body surface area.

**Table 2:** Hemodynamic data

		**T**_**0**_	**T**_**2**_	**T**_**6**_	**T**_**12**_	**T**_**24**_	**T**_**48**_	**T**_**72**_
**Heart rate, min**^**-1**^		89 ± 17	88 ± 19	89 ± 19	86 ± 20	88 ± 17	84 ± 15	88 ± 16
**MAP, mmHg**		106 ± 14	106 ± 11	104 ± 14	106 ± 12	110 ± 13	114 ± 13*	112 ± 13
**CVP, mmHg**		12 ± 4	12 ± 3	13 ± 4	11 ± 4	12 ± 6	13 ± 3	12 ± 4
**ITBV, ml**		1638 ± 330	1711 ± 405	1703 ± 391	1708 ± 301	1682 ± 311	1739 ± 359	1755 ± 338
**SVR, dyn s cm**^**-5**^		923 ± 295	902 ± 249	891 ± 207	926 ± 209	980 ± 249	1016 ± 251	973 ± 237
**ICP, mmHg**		7 ± 4	8 ± 4	7 ± 3	9 ± 3	8 ± 5	8 ± 4	6 ± 4
**Blood flow velocity, cm s**^**-1 **^**(median/range)**	**ICA right**	63 (22 to 100)				74 (31 to 145)	68 (30 to 185)	60 (20 to 129)
	**ICA left**	75 (33 to 101)				68 (35 to 147)	77 (33 to 200)	66 (20 to 145)
	**MCA right**	129 (45 to 185)				137 (51 to 223)	126 (40 to 211)	114 (43 to 164)
	**MCA left**	110 (27 to 224)				134 (61 to 280)	140 (76 to 224)	129 (35 to 272)
**Hemoglobin, g l**^**-1**^		11.2 ± 1.1	11.1 ± 1.1	11.1 ± 1.3	11.0 ± 1.4	11.5 ± 1.3	11.2 ± 1.3	10.7 ± 1.5
**Norepinephrine, μg kg**^**-1 **^**min**^**-1**^		0.61 ± 0.51	0.59 ± 0.52	0.64 ± 0.61	0.69 ± 0.82	0.66 ± 0.79	0.49 ± 0.44	0.44 ± 0.35

Data are presented as mean ± SD if not stated otherwise. MAP, mean arterial pressure; CVP, central venous pressure; ITBI, intrathoracic blood volume index; SVRI, systemic vascular resistance index; ICP, intracranial pressure; ICA, internal carotid artery; MCA, middle cerebral artery.

*P <0.05 versus time (T)_0._

From the 20 patients included, a total of 136 sets of CO measurements were available for comparison of TPCO and PCCO. Due to technical problems, CO data pairs were available only for the first 24 hours in one patient, and in two patients only for the first 48 hours. TPCO ranged from 5.2 to 14.3 l · min^-1^ and PCCO ranged from 5.0 to 14.4 l · min^-1^ (Table 3).

**Table 3:** Statistical analysis of pulse wave-derived cardiac output measurements and of the reference technique

	**T**_**0**_	**T**_**2**_	**T**_**6**_	**T**_**12**_	**T**_**24**_	**T**_**48**_	**T**_**72**_	**T**_**all**_
**TPCO, l · min**^**-1**^	8.8 ± 2.4	8.8 ± 2.1	8.6 ± 2.0	8.6 ± 1.9	8.3 ± 1.8	8.3 ± 1.7	8.6 ± 2.0	8.5 ± 2.0
**CV TPCO, %**	2.2	2.1	2.5	2.0	2.1	3.0	2.4	2.3
**CE TPCO, %**	1.2	1.2	1.4	1.1	1.2	1.6	1.2	1.3
**Precision TPCO, %**	2.4	2.4	2.7	2.2	2.3	3.3	2.5	2.6
**PCCO, l min**^**-1**^	8.8 ± 2.4	8.8 ± 2.5	8.6 ± 2.0	8.7 ± 2.0	8.3 ± 1.5	8.7 ± 1.8	8.6 ± 2.3	8.6 ± 2.0
**Bias, l min**^**-1**^	0.0 ± 0.4	0.1 ± 0.9	0.0 ± 0.7	-0.1 ± 0.9	0.1 ± 0.7	-0.4 ± 0.9	-0.1 ± 0.9	-0.03 ± 0.8
**Limits of agreement, l min**^**-1**^	0.9	1.8	1.4	1.8	1.4	1.8	1.7	1.6
**PE PCCO, %**	9.9	20.0	16.1	20.5	17.2	21.8	19.8	18.4
**Precision PCCO, %**	8.9	19.6	15.3	20.2	16.7	20.9	19.2	17.8

Data are presented as mean ± SD if not stated otherwise. T, time; TPCO, transpulmonary cardiac output; PCCO, pulse wave-derived cardiac output; CV, coefficient of variation; CE, coefficient of error; PE, percentage error.

**Figure 2:**
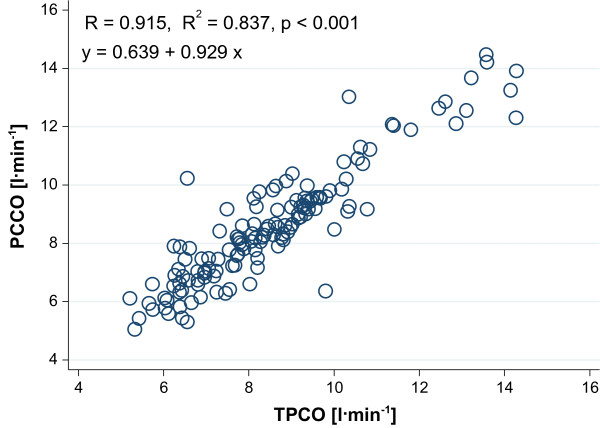
Linear correlation analysis of the relationship between transpulmonary thermodilution cardiac output (TPCO) and pulse-contour derived cardiac output (PCCO) for all data.

**Figure 3:**
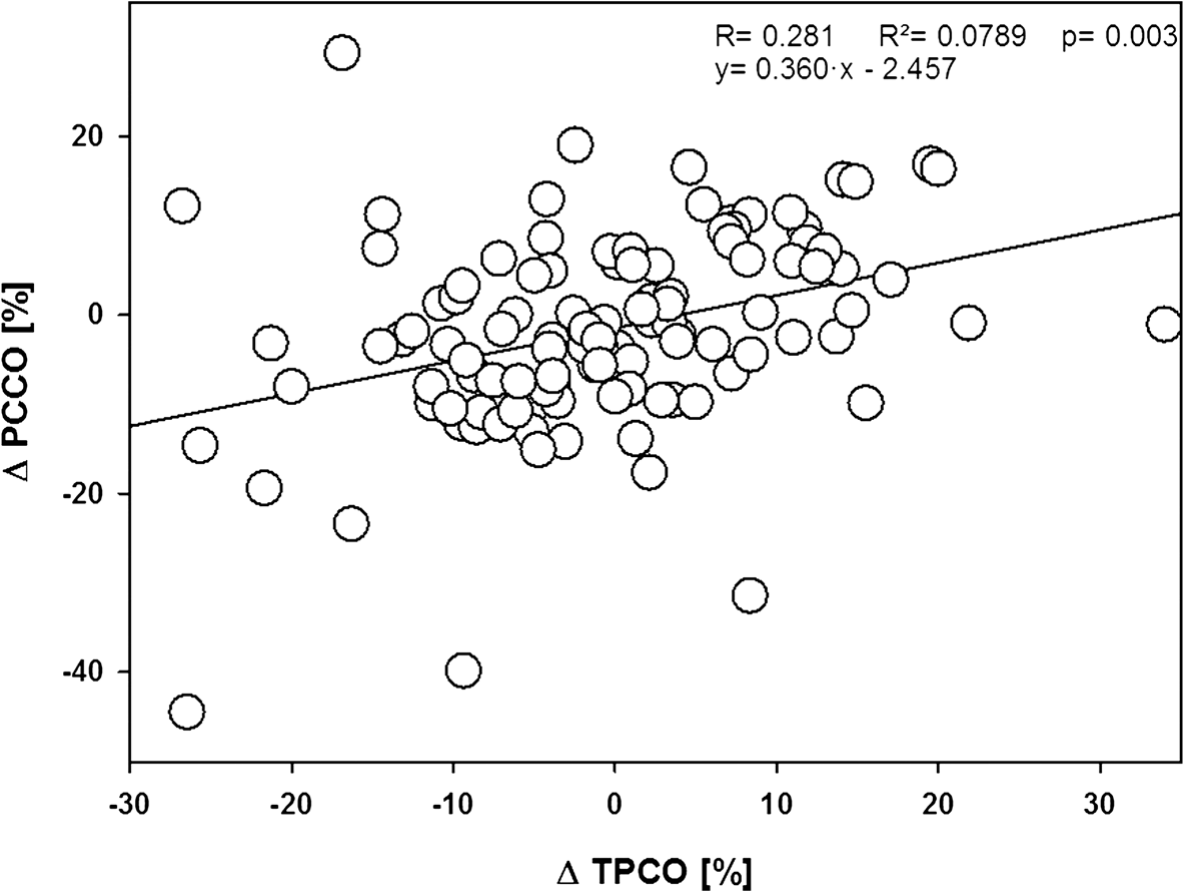
Linear correlation analysis of the relationship between the percentile changes as registered by transpulmonary thermodilution cardiac output (TPCO) and the changes indicated by pulse-contour derived cardiac output (PCCO) measurements between each time point.

Linear correlation analysis showed acceptable correlation between TPCO and PCCO only for absolute values (Figure 2). The correlation for the percentage changes in CO between each time point was poor (Figure 3).

**Figure 4:**
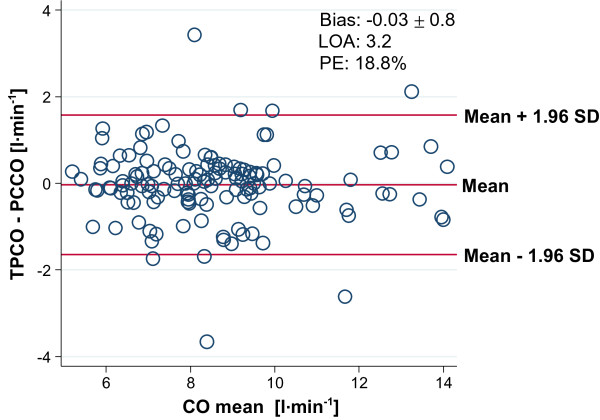
**Bland-Altman analysis for cardiac output (CO) measurements by transpulmonary thermodilution cardiac output (TPCO) and by pulse-contour derived cardiac output (PCCO) for all data.** Limit of agreement (LOA) is defined as the difference between the upper and the lower level of the limits of agreement (−1.64 − 1.58 l · min^-1^). LOA. PE, percentage error.

A detailed statistical analysis of the comparison of TPCO and PCCO measurements is shown in Table 3, including the precision of the reference technique. For all data pairs, Bland-Altman analysis revealed a bias of −0.03 ± 0.82 l · min^-1^ and a limit of agreement of 1.62 l · min^-1^ (Figure 4), resulting in an overall percentage error of 18.8%. The precision of all PCCO measurements was 17.8% (Table 3).

**Figure 5:**
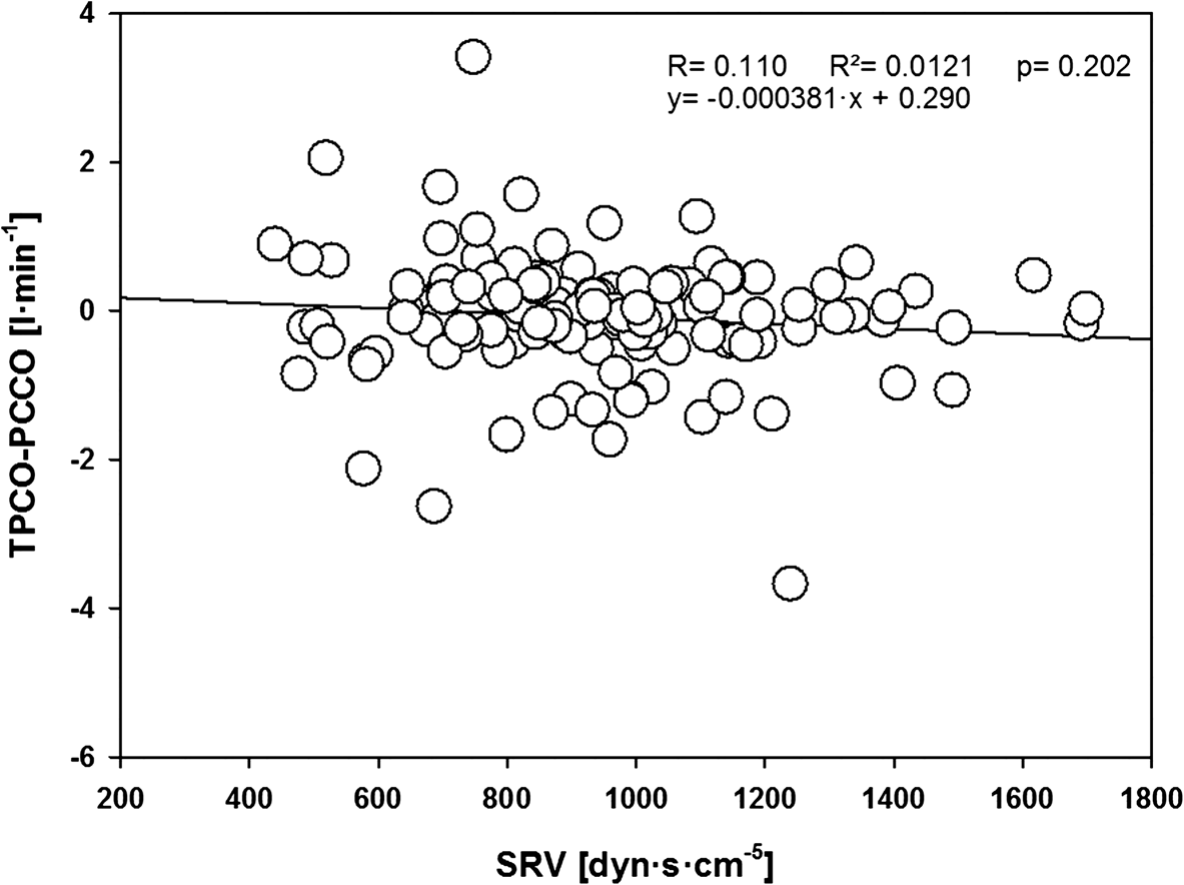
Linear correlation analysis of the relationship between systemic vascular resistance (SVR) and transpulmonary thermodilution cardiac output (TPCO)-pulse-contour derived cardiac output (PCCO) for all data.

Linear correlation analysis revealed no correlation between the bias between TPCO-PCCO and SVR (Figure 5).

**Figure 6:**
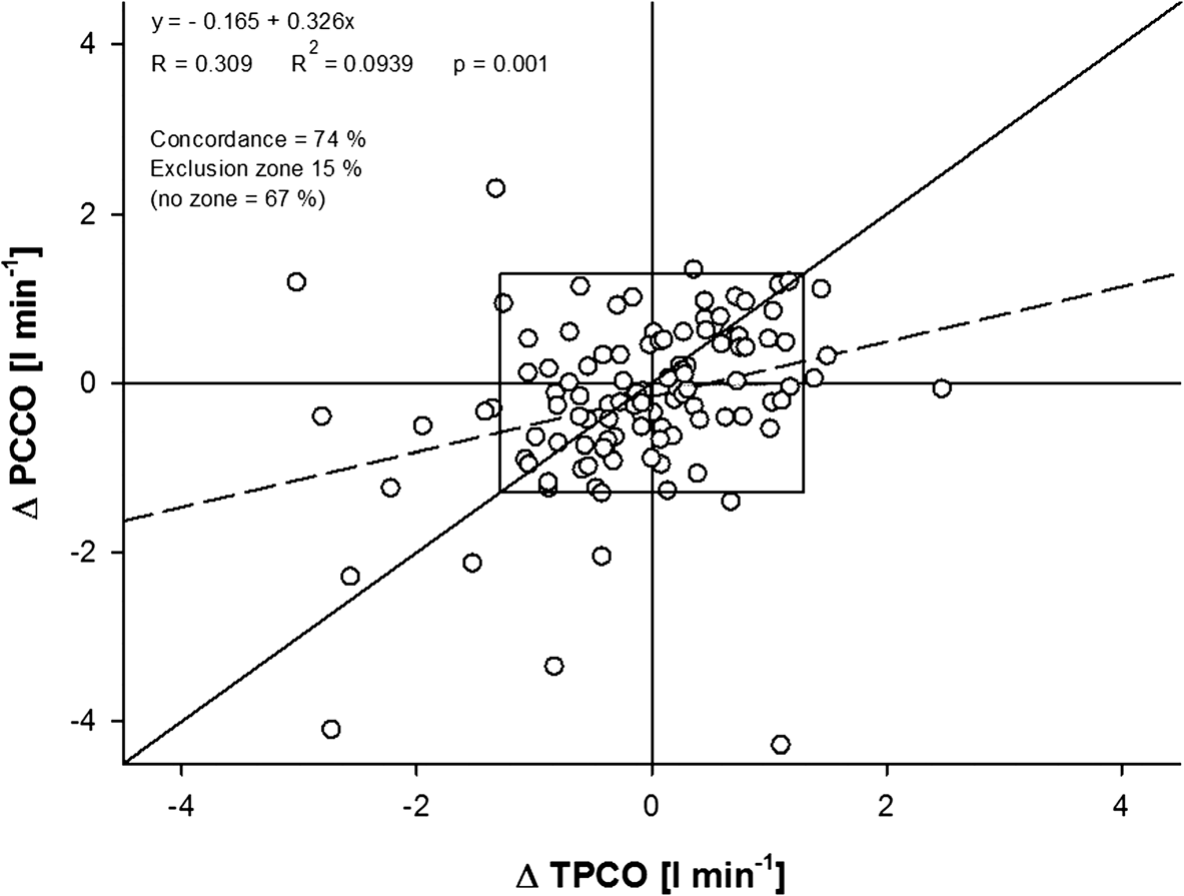
**Concordance rates with and without the exclusion zone of 15%.** TPCO, transpulmonary thermodilution cardiac output; PCCO, pulse-contour derived cardiac output.

**Figure 7:**
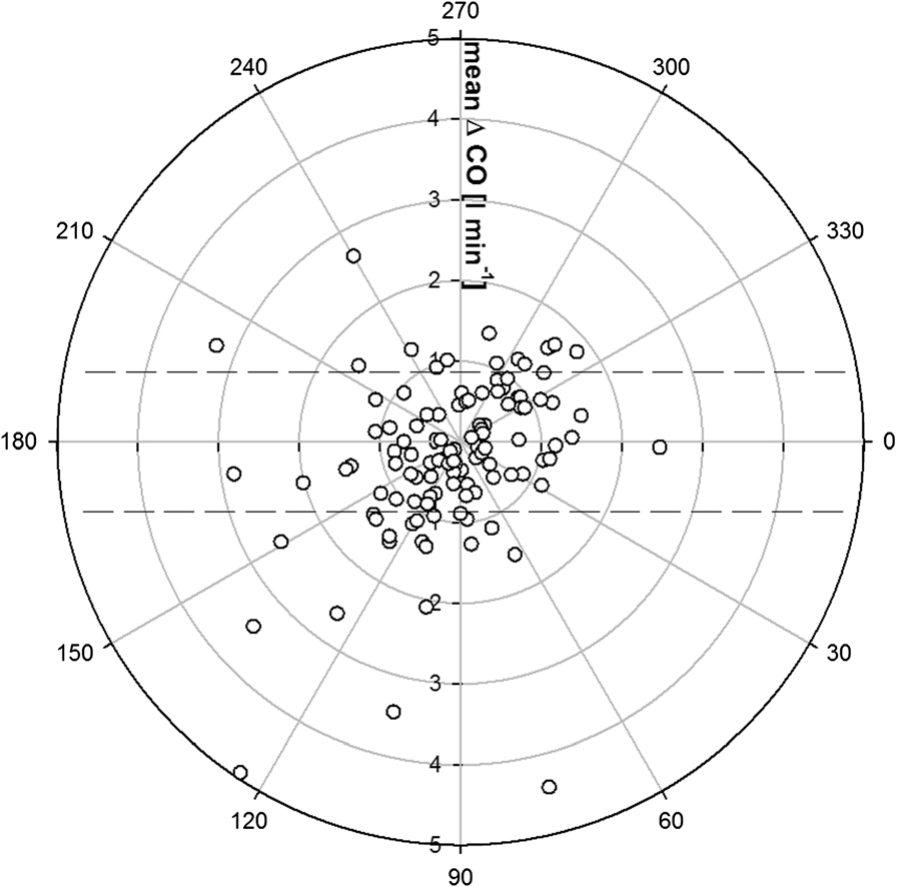
**Polar plot with a 10% band as the limit of good agreement.** CO, cardiac output.

Trending ability of PCCO as analyzed by the concordance method is shown in Figure 6. It reveals a concordance rate of 74% with an exclusion zone of 15%. Without applying an exclusion zone the concordance decreased to 67%. Figure 7 shows the results of polar-plot analysis. In our study (mean CO = 8.6 l min^-1^) only 69.6% of the data lie within the 10% band, suggesting poor trending ability.

PCCO measurements using the PiCCO technology is a well-established and increasingly popular method of minimally invasive hemodynamic monitoring. The validity of PCCO measurements has been studied extensively [3,5,6,9]. However, most of these validation studies have been performed in cardiac surgical patients requiring only low-to-moderate doses of vasoactive support [3,5,6,9]. In contrast we analyzed the validity and accuracy of PCCO measurements in a setting outside of cardiac surgery and as recently demanded [31], in a select patient group under extreme cardiocirculatory conditions, that is, in neurosurgical patients requiring high-dose vasopressor support for the prevention/treatment of DCI due to SAH.

The results of our study demonstrate that in this patient population, CO measurements by calibrated arterial pulse-contour analysis revealed a percentage error of approximately 20% for agreement with the reference technique. Likewise, detailed statistical analysis demonstrated the precision of PCCO measurements to be below 20%. These results allow the consideration of the technology of PCCO measurements as clinically interchangeable with the reference technique, at least in this specified patient population.

## Discussion

Until today there is no consensus on the most appropriate statistical methodology for the validation of continuous CO monitoring-techniques [32]. In the first step, we used the method introduced by Critchley and Critchley [11] for the analysis of the agreement between PCCO measurements and TPCO as the reference technique. These authors originally suggested that the alternative method should have an equivalent precision to the chosen reference method to postulate interchangeability of the two methods. Critchley and Critchley proposed that the reference method has to yield a precision of approximately 20%. Hence (see also Equation 4 in Materials and methods), the percentage error derived from the Bland-Altman analysis should be <28.3% (or, as suggested by several authors, <30%).

As recently discussed, the rigid application of the ±30% cutoff for the percentage error can potentially hide important information as two separate levels of precision contribute to it, of which only the combination adds up to the cutoff value [12,25]. Hence, a true interpretation of the total percentage error is only possible if the precision of each method is reported separately. In our study, both the precision of the TPCO (<3%) and the PCCO measurements (<18%) was lower than the precision of 20% as originally suggested by Critchley and Critchley, resulting in an overall percentage error <20%.

Analyzing the trending accuracy of the test method compared with the reference method revealed a concordance rate of 74% with a 15% exclusion zone. According to a recent overview by Critchley *et al*. [16], hence, the trending ability of the PCCO measurement can be considered poor. Because there is no consensus on an appropriate exclusion zone, and several different exclusion zones have been applied in the literature [14,23,33,34], we also performed the concordance analysis using exclusion zones of 0%, 5% and 10%, respectively. This allowed the inclusion of substantially more data points into the analysis. However, narrowing the exclusion zone did not result in an improvement of concordance as expressed by concordance rates of 67%, 68% and 63%, most probably due to the fact that by this approach more central data points, and hence, more statistical noise, were included in the analysis. It has to be noted that the spread of percentage changes also affects the concordance rate. In our study, the majority of ΔCO values were rather small, which may have resulted in skewed distribution of the data points and might be associated with false estimation of the concordance rate. Therefore, we additionally performed a polar-plot analysis as recently suggested by Critchley *et al*. [16] that allows us to account for both the magnitude of the underlying CO changes and the degree of agreement. Also this analysis yielded an insufficient trending ability for the PCCO technique.

Owing to the underlying calculation algorithms, PCCO monitors have to be regularly recalibrated to obtain stroke volume by an independent reference method [35-37]. However, as yet no consensus exists on the appropriate recalibration intervals for different patient populations. In our study, no major changes in either cardiac preload (as reflected by ITBV) or vascular tone (as roughly reflected by SVR) occurred throughout the observation period. In this situation, even recalibration intervals of up to 24 hours were not associated with a significant loss of accuracy for PCCO measurements. On the other hand, it is well-known that PCCO measurements in situations of hemodynamic instability with acute changes of vascular tone are often unreliable unless short recalibration intervals down to 1 hour are employed [35-37]. Of note, more frequent recalibrations also allow updating of relevant hemodynamic information drawn from other thermodilution-derived variables [38].

Another interesting finding of our study was that the accuracy of PCCO measurements was not influenced by SVR, which is in contrast to the cardiac output measurements derived by auto-calibrating pulse-contour analysis, as recently reported by us using the same study design, and by other groups [20-22,25].

The present study has several limitations. Only a highly selected patient-collective was included in the study, so that extrapolation of the results to other patient populations is hardly possible. Moreover, all of our patients exhibited normal to supranormal cardiac outputs. The accuracy of PCCO measurements in patients with low cardiac outputs could therefore, not be analyzed and remains to be investigated. Finally, despite the high-dose vasopressor therapy all patients were in a hemodynamically stable state in which no major changes of cardiac preload or vasotonus occurred.

## Conclusions

In neurosurgical patients requiring extensive vasoactive support, CO values obtained by PCCO showed a percentage error of <20% for the agreement with TPCO measurements as the reference technique. This error is commonly regarded as a criterion for method interchangeability. The precision of calibrated CO measurements was clinically appropriate and independent of SVR. However, owing to the poor trending ability of the PCCO device, caution is warranted when basing hemodynamic management solely upon the results of the pulse-contour analysis rather than performing frequent TPCO measurements.

## Key messages

•In patients requiring extensive vasoactive support for the treatment of DCI, PCCO measurements showed a percentage error <20% to show agreement with the reference technique.

•In comparison to TPCO, the precision of PCCO measurements was appropriate.

•The accuracy of PCCO measurements was independent of systemic vascular resistance.

•In our patients, PCCO measurements did not reliably track CO changes.

## Abbreviations

BSA: Body surface area; CE: Coefficient of error; CO: Cardiac output; CPP: Cerebral perfusion pressure; CV: Coefficient of variation; CVP: Central venous pressure; DCI: Delayed cerebral ischemia; H&H: Hunt and Hess grade; HR: Heart rate; ICA: Internal carotid artery; ICP: Intracranial pressure; ITBV: Intrathoracic blood volume; MAP: Mean arterial pressure; MCA: Middle cerebral artery; PCCO: Pulse-contour derived cardiac output; PE: Percentage error; SAH: Subarachnoid hemorrhage; SVR: Systemic vascular resistance; TPCO: Transpulmonary thermodilution cardiac output.

## Competing interests

All involved authors declare that they have no competing interests.

## Authors’ contributions

SR conceived of the study and - together with SM - developed its design, performed the hemodynamic measurements, the data acquisition, the statistical analysis and wrote the manuscript. MC, CS, MF, TS, MR, AH, RR and GM participated in the study design and/or hemodynamic measurements. All authors read and approved the final manuscript.
